# Electrospun Polymeric Fiber Systems Inoculated with Cyanoacrylate Tissue Adhesive: A Novel Hemostatic Alternative during Open Surgery

**DOI:** 10.3390/ma17174318

**Published:** 2024-08-30

**Authors:** Victor P. Tosa, Alexandru Ilie-Ene, Septimiu C. Tripon, Amalia Mesaros, Radu Fechete, Nicoleta Tosa, Alexandra Csapai, George C. Dindelegan, Catalin O. Popa

**Affiliations:** 1 Materials Science and Engineering Department, Technical University of Cluj-Napoca, 103-105 Muncii Ave., 400641 Cluj-Napoca, Romania; amalia.mesaros@chem.utcluj.ro (A.M.); radu.fechete@phys.utcluj.ro (R.F.); alexandra.csapai@stm.utcluj.ro (A.C.); catalin.popa@stm.utcluj.ro (C.O.P.); 2 Department of Surgery, Iuliu Hatieganu University of Medicine and Pharmacy, 8 Victor Babes Street, 400012 Cluj-Napoca, Romania; george.dindelegan@umfcluj.ro; 3 National Institute for Research and Development of Isotopic and Molecular Technologies, 67-103 Donat Street, 400293 Cluj-Napoca, Romania; septimiu.tripon@itim-cj.ro (S.C.T.); nicoleta.tosa@itim-cj.ro (N.T.)

**Keywords:** electrospinning, polylactic acid, wound dressing, tissue adhesive

## Abstract

Natural-based and synthetic tissue adhesives have attracted extensive attention in the last two decades for their ability to stabilize uncontrolled bleeding instances. However; these materials present several drawbacks during use that scientists have tried to minimize in order to optimize their usage. This study comprises the development of a novel wound dressing, combining the excellent properties of polylactic acid (PLA) non-woven textile, as substrate, obtained through electrospinning, and a cyanoacrylate-based (CA) tissue adhesive, for rapid hemostatic action. Thus, the fabrication of electrospun PLA membranes at three different PLA concentrations, the design and manufacturing of the support system and the production of surgical patches were carried out. SEM and FT-IR methods were employed for analyzing the morphology as well as the indicative markers for the shelf life evolution of the obtained patches. PLA fibers with well-defined structures and a mean diameter varying between 4.6 and 7.24 μm were obtained with the increase of the concentration of the PLA solutions. In vivo tests on a rat model as well as peeling tests for good patch adhesion on liver fragments harvested from the test animals, with a limit for the strength of the liver tissue of 1.5 N, were carried out. The devices exhibited excellent adhesion to the parenchymal tissue and a long enough shelf life to be used with success in surgical procedures, also facilitating prompt hemostatic action.

## 1. Introduction

Surgical procedures globally reached approximately 300 million in 2023 [1,2,3], with projections anticipating a rise to 375 million by the decade’s end [4]. Consequently, there has been a notable increase in wounds associated with uncontrolled bleeding and infections [5]. Achieving hemostasis during surgical interventions is paramount, especially in trauma settings [6,7,8]. Established techniques such as sutures, cauterization and hemostatic materials are currently relied upon by surgeons [9,10,11]. Cyanoacrylate tissue adhesives, a category of synthetic topical hemostats, offer advantages including robust wet adhesion, cost efficiency, antibacterial properties and favorable cosmetic outcomes [11,12,13,14,15]; they realize rapid hemostasis onto a wound surface in dozens of seconds by an airflow-directed in situ electrospinning method [16] as well as improving the quality of the in situ dural closures after neurosurgery [17]. However, the rapid onset of anionic polymerization poses a challenge, necessitating the use of inhibitors [18]. Sulfur dioxide, among others, serves as an inhibitor; however, its concentration must be carefully controlled to avoid adverse reactions [19,20]. Polymer fiber-based membranes present a potential solution for enhancing an effective delivery of cyanoacrylates using capillary mechanisms such as the ones specific to paper microfluidics. Aerogels produced from marine polymers, such as chitosan and alginate [21], fish biowaste gelatin coated phosphate-glass fibers [22], natural electrospun polymers [23], physically blended antibacterial triclosan (Tri) agent with poly(lactic-co-glycolic acid) (PLGA) and poly(ethylene oxide) (PEO) polymers [24], as well as polyvinylpyrrolidone (PVP) and metronidazole dispersed in polycaprolactone (PCL) as a slow release core [25] were use to produce fibers by different types of electrospinning processes for wound healing applications. Polylactic acid (PLA) is a notable polymer due to its low toxicity, easy processing, biocompatibility, bio-degradability, hydrophobicity and compostability [26,27,28,29,30,31,32]. However, producing PLA substrates with paper-like microfluidic characteristics is challenging through conventional methods. Electrospinning emerges as a standout method for fabricating PLA non-woven fiber structures [33]. This technique involves drawing fibers from a spinnable source by applying a strong electric field [34]. A conventional electrospinning setup includes a high-voltage power supply, a grounded conductive collector and a syringe with a spinneret [35]. Upon application of a high electrical charge, a “Taylor cone” forms, initiating ejection of a fine polymer jet directed towards the collecting substrate [36]. The interplay of forces induces self-repulsion of the polymer, leading to the deposition of randomly packed fibers on the substrate [37]. The fabrication of electrospun PLA [38] and the application of cyanoacrylate tissue adhesives [39,40,41,42] are well-documented in the scientific literature. However, the combined use of these two materials to enhance hemostatic efficacy remains untested. In this paper, the fabrication process of a novel wound dressing device, comprised of a PLA non-woven structure inoculated with medical grade cyanoacrylate tissue adhesive for rapid hemostatic action, is being presented. SEM and FT-IR analysis was employed analyzing the structure of the obtained patches. In vivo testing on rat test subjects as well as peeling tests for patch adhesion on liver fragments harvested from test animals further support the novel fabricated wound dressing’s functionality.

## 2. Materials and Methods

### 2.1. Manufacturing of Electrospun Substrate

A high voltage source in combination with a syringe pump and a static collector, in a custom-made setup, were used for the manufacturing of non-woven fibrous structure membranes through the electrospinning process. PLA powder (average Mw ≈ 60,000 g/mol, purchased from Sigma Aldrich Ltd., St. Louis, MO, USA) was dissolved in chloroform (EMSURE, ACS, ISO, Merck, Darmstadt, Germany), assisted by ultrasonication, to form solutions with weight percentage concentrations of 6%, 8% and 10%. These solutions were electrospun at ambient temperature using the following setting parameters: 50 kV voltage, needle to collector plate distance of 105 mm and a flow rate of 0.075 mL/min from 10 mL syringes with needle and extension scale (Jiangsu Zhengkang Medical Apparatus Co., Ltd., Changzhou, China).

### 2.2. Manufacturing of the Wound Dressing Support and Tensile Strength Test Systems

**Figure 1:**
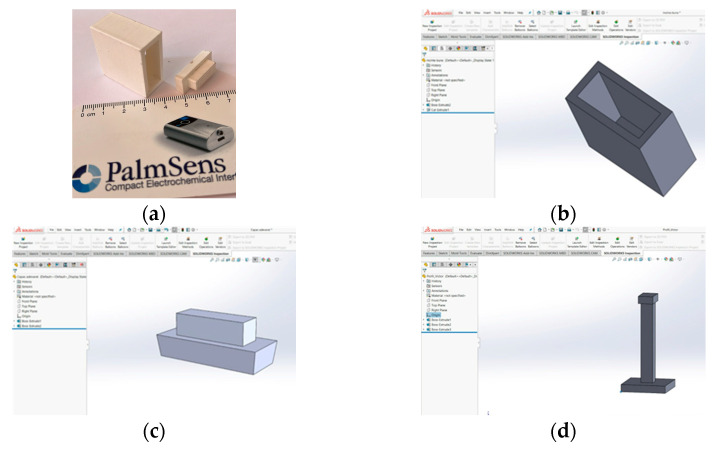
PLA systems of: wound dressing support—physical aspect (**a**); 3D design of Box (**b**); Lid (**c**) and 3D design of grips for tensile strength testing (**d**).

A Crealty Ender 5 3D printer (Shenzhen Creality 3D Technology Co., Ltd., Shenzhen, China) was used with a 1.75 mm high performance PLA filament (VerbatimTM- Mitsubishi Kagaku Media Co., Ltd., Tokyo, Japan) to manufacture a support (PLA box & lid) for handling and storing wound dressings (Figure 1a–c), as well as specially designed PLA grips for tensile strength testing (Figure 1d) systems. The design was created using SolidWorks 3D CAD software (EducationEdition 2019–2020, Dassault Systèmes, Vélizy-Villacoublay, France) (Figure 1b–d).

### 2.3. Production of Surgical Patches

First, a protective system consisting of a sterile vacuum bag with an inserted silicone sleeve sealed with Buildfix Pro DC hybrid composite (P.L. Superior Dental Materials GmbH, Hamburg, Germany) was created. Then, the PLA membranes were placed inside the PLA box, which in turn was placed inside the bag and fixed with the help of the silicone sleeve.

After the bag was sealed and additionally vacuumed, the PLA electrospun substrates within the support systems underwent a washing procedure with sulfur dioxide (SO_2_) gas, obtained by mixing hydrochloric acid (HCl) and sodium sulfite (Na_2_SO_3_). The SO_2_ gas was designated to act as a polymerization inhibitor for the cyanoacrylate tissue adhesive, which was to be inoculated in the PLA substrate in the next step of the production. The transfer of the IFA Bond from the original container into the syringe, with which to inoculate the PLA dressing during the adhesive deposition stage, was done inside the glovebox under nitrogen gas in the presence of silica gel to ensure inert/water-free working conditions. Once the steps to be followed were established and completed, approximately 0.1 mL of tissue adhesive was delivered inside the sealed bag on each PLA substrate, which has an area of approximately 150 mm^2^.

### 2.4. Characterization and Functional Testing

In order to assess the evolution of the PLA substrates, which were obtained at the PLA concentrations of the solutions presented earlier, qualitative analysis was employed in the form of SEM imaging. Before imaging, the samples were coated with a 10 nm gold layer by sputtering at a low vacuum of 10 mbar (10^3^ Pa) to increase the contrast in the recorded secondary electron images. Images of the substrate fiber matrix morphology were recorded using a Hitachi SU 8230 SEM system (Hitachi High-Tech Corporation, Tokyo, Japan) operating at accelerating voltages of 10 kV and magnifications ranging from 100× to 5000×. The fiber diameter measurements were done on 100 fibers in at least five different positions along each fiber using the free access ImageJ program. The collected data were then used to plot the histogram.

For the purpose of determining the evolution of the shelf life of the active wound dressing placed inside the support systems, FT-IR analyses were performed using a JASCO FT/IR-8X FT-IR system (Jasco Corporation, Tokyo, Japan), using a Jasco software version2.01.02, operating at a resolution of 4 cm^−1^ with the number of scans equal to 64. Spectral range/horizontal view range was between 350–4000 cm^−1^.

In vivo testing was employed in the form of hepatic resections on a rat model, in order to observe the efficacy of this novel hemostatic delivery system. These tests were conducted with the approval of the Ethics Committee of “Iuliu Hatieganu” University of Medicine and Pharmacy, Cluj-Napoca, Romania, Nr. 377/25.08.2023.

Also, in order to assess the adhesion capabilities of the wound dressing to parenchymal tissue, peeling tests were performed on freshly harvested rat liver with a bound patch, using a Zwick/Roell Pro Line BL-GRP005K 5kN machine (Zwick/Roell Company, Ulm, Germany) using specially designed PLA grips (Figure 1d). The tests were qualitative, to prove the adhesivity of the patches to the liver, in this case on a rat model. The experimental setup was conceived in order to check if the surgical patches could peel off after being glued to the liver.

## 3. Results and Discussion

### 3.1. Scanning Electron Microscopy

**Figure 2:**
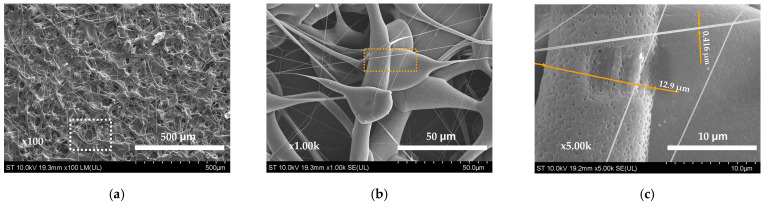
SEM images of electrospun PLA membrane obtained from a 6% PLA solution: (**a**) at 100× magnification; (**b**) detail at 1000× magnification; (**c**) marked detail at 5000× magnification.

In the production of PLA substrates, solutions with varying PLA concentrations (6%, 8% and 10%) in chloroform were utilized. The electrospinning process allows the formation of PLA fibers from solutions under the conditions in which the solvent vaporizes and the dissolved material from the solutions is restructured in the form of a fiber. Electrospinning yielded substrates with distinct textures and appearances, as demonstrated by SEM images. Morphological analysis, including examinations of pore size and distribution on the fibers, was conducted for all three concentrations. The substrate with a 6% PLA exhibited a rather compact fibrous membrane that lacked uniformity, particularly in terms of fiber diameters (Figure 2a). Higher magnifications (1000×, 5000×) revealed significant beading throughout the structure. Fiber diameter measurements ranged from 0.4 to 12.9 microns, with a mean diameter of 6.6 microns. The larger fibers displayed microporosity and larger defects (Figure 2b,c).

At lower concentrations, due to the large volume of solvent that has to evaporate, the amount and density of the useful material will be lower, so that structures with irregular shapes and sizes will be formed, either very thin or very large. At the same speed of pumping for the solutions in the electrospinning process, there can also be an incomplete evaporation of the solvent at the level of the “cone”, so that drops of preconcentrated solution are splashed towards the collector and the evaporation of the solvent occurs only after the drop falls on the surface of the collector. Finally, the appearance of the material deposited on the collector will be that of a network of fibers with extremely variable widths along their entire length. The overall aspect of the obtained morphology prompted an increase in PLA concentration.

**Figure 3:**
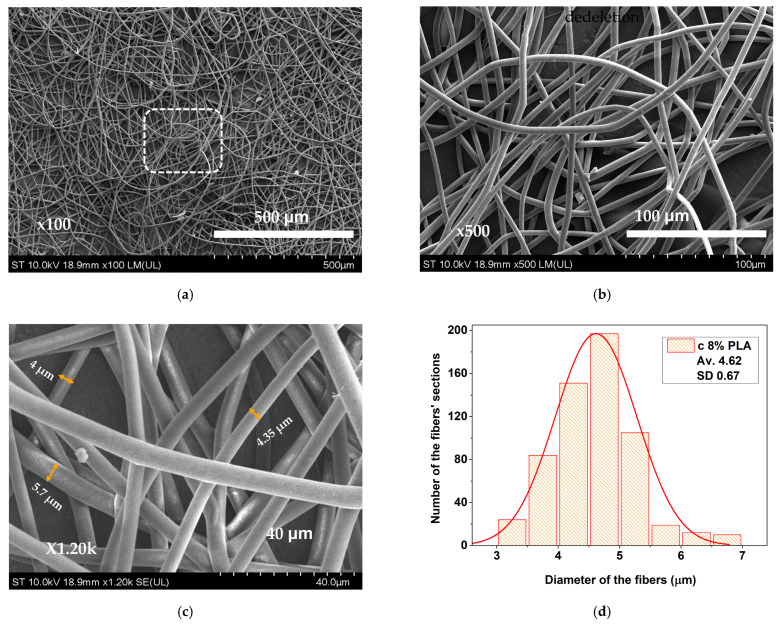
SEM images of electrospun PLA membrane obtained from an 8% PLA solution: (**a**) at 100× magnification; (**b**) detail at 500× magnification; (**c**) marked detail at 1200× magnification; (**d**) histogram.

At higher concentrations, the lower volume of solvent that needs to evaporate will cause a larger and denser base of useful material to be formed at the “cone” level, which will allow for the constant formation of much more uniform-looking fibers. At 8% PLA concentration, substantial reduction in beading was observed, forming a well-defined fibrous structure (Figure 3a,b). Additionally, the higher PLA content led to an increased density and to a higher surface microporosity of fibers (Figure 3c). The mean fiber diameter decreased to approximately 4.62 µm, ensuring greater uniformity (Figure 3d).

**Figure 4:**
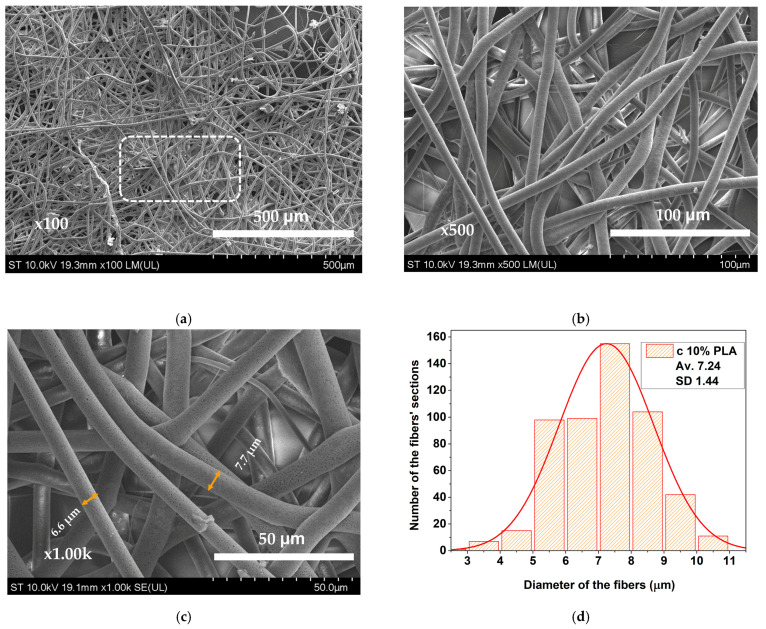
SEM images of electrospun PLA membrane obtained from a 10% PLA solution: (**a**) at 100× magnification; (**b**) detail at 500× magnification; (**c**) marked detail at 1000× magnification; (**d**) histogram.

Advancements were further observed in the 10% PLA solution, evidenced by the formation of smoother fibrous structures (Figure 4a–c). SEM imaging showed a well-defined fiber membrane with higher density than the 8% solution ones. Fiber diameter ranged from 2.8 to 10.7 microns, with a mean diameter of 7.24 microns (Figure 4d). Microporosity, consistently present throughout the fibers at higher magnifications (Figure 4c), proved an efficient solvent evaporator, leading to enhanced membrane quality. Detachment of the substrates from the collector plate was notably easier for the 10% PLA solution, which represents an advantage in the reproducibility of experiments. The smooth surface combined with the desired mechanical properties confirmed that the 10% PLA concentration yielded membranes with the best characteristics among the studied ones. Consequently, these were employed in fabricating the necessary wound dressing devices that were subsequently tested in vivo.

**Figure 5:**
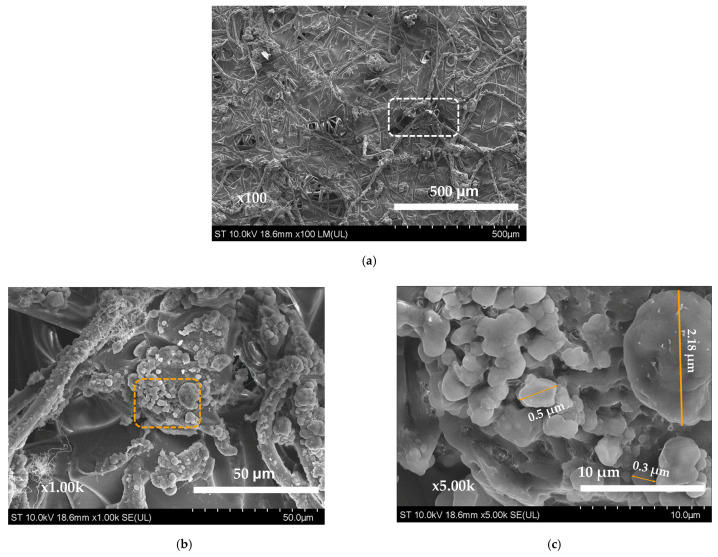
SEM image of electrospun PLA fiber matrix for c = 10%, inoculated with CA tissue adhesive: (**a**) at 100×; (**b**) detail at 1000× magnification; (**c**) detail at 5000× magnification.

A significant development was the prolonged active state of the cyanoacrylate in the wound dressing, due to the introduction of SO_2_ gas as a polymerization inhibitor. One patch from the batch was selected randomly for subsequent SEM imaging analysis (Figure 5a). While preparing the sample for SEM analysis, it was revealed that hours after exposure to atmospheric conditions a part of the adhesive retained its unpolymerized state, highlighting the effectiveness of SO_2_ as a reaction inhibitor. SEM analysis further showcased a visible bond between the substrate and tissue adhesive. Higher magnifications revealed numerous small polymerized cyanoacrylate nuclei (Figure 5b,c), with diameters ranging from 3 to 500 nanometers.

This suggests that the micropores on the fibers constitute attachment sites for the cyanoacrylate during its early polymerization. As a result, when the polymerization process continues, these attached nuclei grow, enhancing the adhesive bond between the substrate and the cyanoacrylate.

### 3.2. FT-IR Analysis

**Figure 6:**
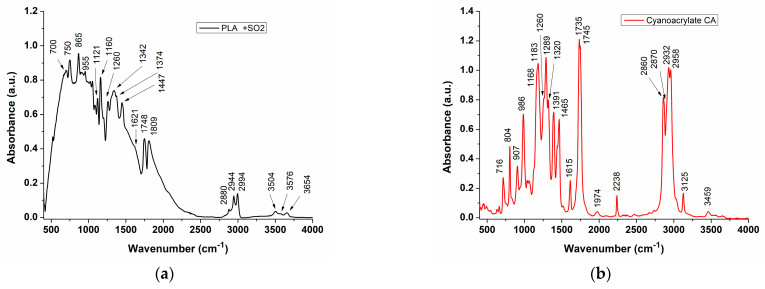
FT-IR absorbance spectra of Electrospun PLA non-woven fibers structure (**a**) and IFABOND cyanoacrylate tissue adhesive (CA) (**b**).

Fourier-transform infrared (FT-IR) spectra of the individual components and the active wound dressings were obtained at various time intervals to monitor the evolution in the structure of patches during and post manufacturing. The chemical structure of the electrospun PLA substrate and IFABOND cyanoacrylate tissue adhesive (CA) [42] were confirmed by the FT-IR spectra in the range of 500–4000 cm^−1^ (Figure 6a,b).

In the FT-IR spectrum of PLA, the peak at 3654 cm^−1^ is assigned to the stretching vibration of the terminal OH group (free), 3504 cm^−1^ for the -OH from the carboxyl terminal group and a doublet band at 2944 cm^−1^ and 2994 cm^−1^ for the asymmetric stretching vibrations of the -CH3 group (split by the vicinity of the methyne group located at the same carbon atom), with the -CH3 symmetric stretching vibration located at 2880 cm^−1^. The -CH (methyne) group displays only one stretching vibration at 2880 cm^−1^, but it is weak and difficult to identify, being overlapped by the -CH3 symmetric stretching vibration peak.

The bands at 1748 cm^−1^ and 1725 cm^−1^ (shoulder, weak) correspond to the stretching vibrations of the unconjugated C=O bond from the ester group and the carboxylate terminal group, respectively. Additionally, peaks at 1621 cm^−1^ and 1813 cm^−1^ indicate stretching vibrations of the C=O bond in the limit structure of the carboxylate ion and the lactone-type cyclic ester created by intermolecular dimerization, respectively.

The peaks at 1447 cm^−1^ and 1374 cm^−1^ correspond to the asymmetric and symmetric bending vibrations of the -CH3 group, respectively. The peak at 1260 cm^−1^ is attributed to the deformation vibration of the -CH group adjacent to the ester and carboxyl terminal groups [43]. The bands at 1160 cm^−1^ and 1121 cm^−1^ are assigned to the asymmetric and symmetric stretching vibrations of C-OH and C-O-C, respectively. The band at 955 cm^−1^ appears due to the out-of-plane deformation of the O-H group [44], while the peak at 865 cm^−1^ is assigned to the rocking vibrations of C-CH3 (methyl-rocking) [45] and C-OO/C-COOH.

The IR spectrum of chloroform contains several bands given by C-Cl and C-H bonds vibrations as follows: 671 (sharp, m-s), 760 cm^−1^ (very large, vs), 1216 (sharp, s), 2400 (sharp, w) and 3019–3040 cm^−1^ (sharp, m) (w-weak, m-medium, s-strong, vs very strong) [46]. Because the IR spectrum of the PLA presents characteristic peaks in both the 600–800 cm^−1^ and 1200–1400 cm^−1^ spectral regions, the first three peaks from the chloroform IR spectrum could not be used as markers to assess the lack or the presence of chloroform in the PLA electrospun tissues. However, the FT-IR data of PLA tissue showed no absorption bands at 2400 and 3040 cm^−1^, indicating no presence of chloroform in the electrospun PLA tissue.

The chemical structure of cyanoacrylate (CA) in its predominant monomer form versus polymer form was confirmed via FT-IR spectrum analysis (Figure 6b).

The spectrum of 2-octyl-cyanoacrylate (CA) exhibits two strong characteristic bands of the octyl-acrylate ester group bounded to the vinylidene group: a doublet at 1735 cm^−1^ and 1745 cm^−1^, corresponding to C=O bond stretching, and 2932 cm^−1^ and 2958 cm^−1^, assigned to asymmetric stretching of the -CH2- and -CH3 groups, respectively. These bands are accompanied by a weak and broad band between 3410–3510 cm^−1^, representing an overtone of the bands at 1735 cm^−1^ and 1745 cm^−1^, as well as a strong to medium band at 2860 cm^−1^ with a shoulder at 2870 cm^−1^, representing symmetric stretching of -CH2- and -CH3 groups as saturated hydrocarbon moieties of the molecule. Additionally, a weak but sharp peak at 3125 cm^−1^ can be attributed to a =CH2 stretching vibration from the vinylidene moiety, indicating an unsaturated hydrocarbon moiety of the molecule.

This in turn can be considered one of the markers of the CA active state monomer and, therefore, highlights the shelf life of the wound dressing. Also, at 3030 cm^−1^, a very weak peak (as a shoulder) attributed to aliphatic -CH asymmetric stretching can be observed, indicating tendencies of CA polymerization, which transform the C=C bond into a C-C bond. At 2238 cm^−1^ is a weak but sharp peak, assigned to a -CN stretching vibration. Its value is higher than normal due to the presence of the octyl acrylate ester group, bounded to the same adjacent sp^2^ carbon atom involved in the vinylidene C=CH2 moiety. The C=C bond from this moiety gives a weak band at 1615 cm^−1^, with voluminous substituents such as cyan (-CN) and octyl acrylate ester lowering its frequency, when both are bonded at the same disubstituted carbon atom.

The peaks at 1465 cm^−1^ and 1391 cm^−1^ (displayed as a doublet) are attributed to the -CH3 asymmetric and symmetric scissoring vibrations, respectively. Also, the shoulders at 1440 cm^−1^ and 1385 cm^−1^, associated to the previous two peaks, represent the -CH2 asymmetric and symmetric scissoring vibrations, the diminished values being attributed to the adjacent esteric oxygen atom. The triplet displaying maxima at 1320, 1289 and 1260 cm^−1^, with intensity ratio of 1:1.4:1, is characteristic to esteric C-O stretching vibrations coupled with C-C vibrations, particularly due to the coupled vibrations of -CH3 and -CH2- groups within the esteric octyl radical [47]. The prominent peak at 1183 cm^−1^, with a shoulder at 1168 cm^−1^, signifies esteric C-O-C antisymmetric and symmetric stretching modes, respectively. The peak at 986 cm^−1^ corresponds to a geminal disubstituted vinylidene moiety C=CH2 bending frequency, while the peak at 907 cm^−1^ indicates the out-of-plane wagging frequency of a terminal =CH2, suggesting the presence of cyanoacrylate (CA) in its monomer form. The peak at 804 cm^−1^ represents the out-of-plane wagging of CH2 from the vinylidene group and the band at 716 cm^−1^ corresponds to -CH2- rocking vibrations, supported by the presence of more than four -CH2- (methylene) groups in the esteric octyl radical chain.

**Figure 7:**
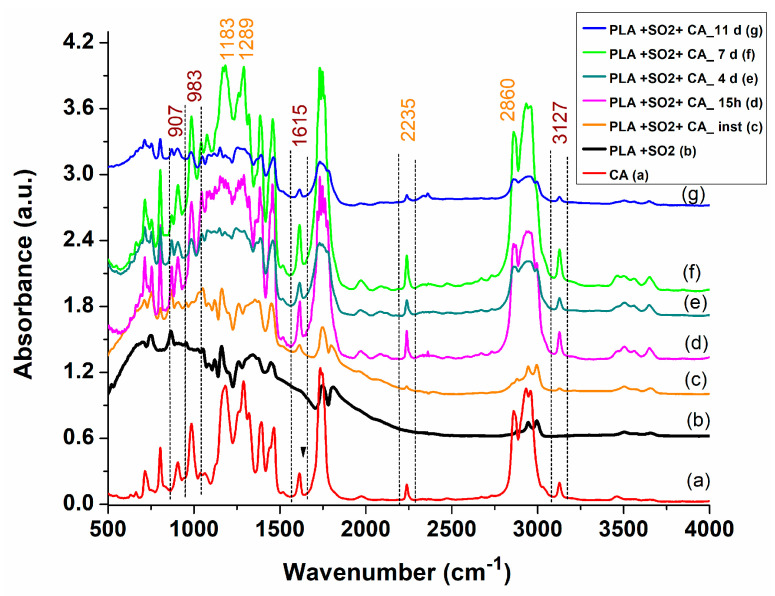
FT-IR absorbance spectra of (a) Cyanoacrylate tissue adhesive (CA); (b) Electrospun PLA non-woven fibrous structure; (c) Electrospun PLA non-woven fibrous structure inoculated with CA, immediately after deposition; (d) Electrospun PLA non-woven fibrous structure inoculated with CA, after 15 h; (e) Electrospun PLA non-woven fibrous structure inoculated with CA, after 4 days; (f) Electrospun PLA non-woven fibrous structure inoculated with CA, after 7 days; (g) Electrospun PLA non-woven fibrous structure inoculated with CA, after 11 days.

Comparison of the FT-IR spectra of PLA substrates inoculated with IFABond CA tissue adhesive, recorded at different times after fabrication, revealed both PLA (Figure 7(b)) and CA (Figure 7(a)) specific bands.

Specifically, bands at 3125 cm^−1^, 1615 cm^−1^, 986 cm^−1^ and 907 cm^−1^ are characteristic of the vinylidene moiety (C=CH2) of the monomeric structure of CA. These bands serve as markers for assessing the maintenance of the wound dressing’s active state. The intensities of these peaks remain consistent in spectra recorded up to a week after fabrication, (Figure 7(c)–(f)) and no longer for those recorded after 11 days (Figure 7(g)), which exhibit new bands indicative of saturated bonds from the polymeric chain, concurrent with a decrease in monomer-specific peaks.

### 3.3. Peeling Test

**Figure 8:**
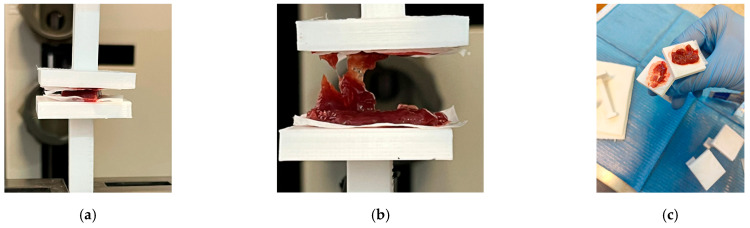
(**a**) Experimental setup for pealing test performed on liver fragments placed between two active wound dressing devices; (**b**) rupture of liver tissue during pealing test displaying excellent adhesion characteristics of the active wound dressing; (**c**) aspect of the PLA grips’ covered pads with liver split fragments at the end of the peeling test.

In order to be able to give the surgeon sufficient information regarding the wound dressings characteristics, peeling tests were carried out on liver fragments harvested from test animals. As depicted in Figure 8a, two identical wound dressing pieces with an area equal to the area of the pads were cut. Each of these pieces was then placed on the pad’s surface of the 3D printed PLA grips, used in the experimental peeling setup. The fixation, adhesion of the PLA based wound dressing to the surface of the PLA grips, was done by means of the IFABOND tissular adhesive [42], with which the electrospun PLA substrate of the device was inoculated and which diffused through capillary action to both sides of the surgical patches. Then, the harvested liver samples (25 mm × 10 mm) were placed then on top of the one of the covered pads of the grips, and the second covered pad was approached until it came into contact with the liver surface. After 5 min of direct contact in a fixed vertical position, the wound dressing (surgical patch) adhered completely to the liver surface as a result of the polymerization of the tissue adhesive CA (Figure 8a). The grips were then fixed between the mounting plates of the device and fixed in position, and the tensile test was performed by moving away the pads of the grips by applying a gradually increased force from 0 to 1.40 N (Figure 8b).

The results for four samples have shown, in all cases, that the patches were so well attached that the liver failed but the adhesive bonding was stable (Figure 8b,c). This behavior indicated a very good adhesion of the surgical patch on the liver surface after 5 min in atmospheric conditions, which was not affected by the forces applied to the liver sample. The results have shown that, during the test, the liver fails, while the patch remains attached. Under these conditions, the value of 1.5 N was found to be a limit for the strength of the liver tissue, as the patch remains glued to the liver (Figure 8c). Because the measured forces, corresponding to the failure of liver tissue (Figure 8b) did not exceed 1.5 N, this indicates that during the surgical procedure the surgeon must apply the wound dressing with great care onto the affected area. The experiment was very useful in validating that following the application of the wound dressing on the affected tissue it can no longer be removed from the surface of the tissue after the tissue adhesive’s polymerization, under conditions in which contact may occur with other types of tissues/organs, other than those on which it was applied. Therefore, after polymerization of the tissue adhesive, the outer surface of the wound dressing, which has not been in contact with the tissue itself, no longer shows adhesion to contact with other tissues or organs.

### 3.4. In Vivo Testing

**Figure 9:**
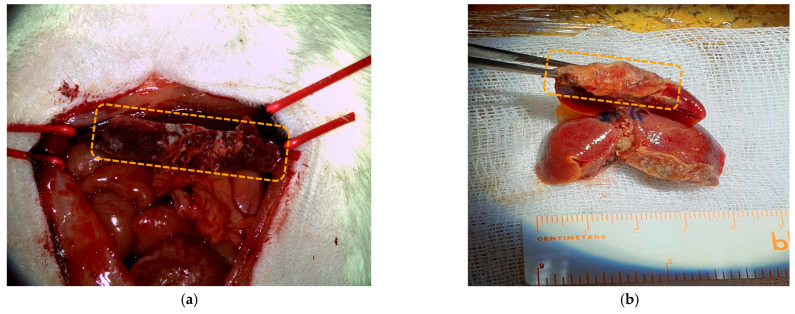
(**a**) Hemorrhage resulted from liver resection treated with PLA wound dressing inoculated with CA tissue adhesive and lobe hemostasis produced after 15 s; (**b**) resorbed patches on the operated liver after 2 months.

Subsequent to the production of the wound dressing devices, they were employed for in vivo testing on animal test subjects, namely, rats. The surgical procedure performed on the test subjects was a liver resection, executed in order to bring about a massive hemorrhage so that the devices’ functionality and efficacy could be tested. As shown in Figure 9a, the resected lobe was wrapped around the damaged tissue where the hemorrhage occurred with the active wound dressing devices. Once the wound dressing was fixated onto the lobe, hemostasis was obtained in an extremely short time span, namely, 15 s. This procedure was performed on multiple subjects in order to assess the success rate of the wound dressing devices, with all of them exhibiting excellent post-operatory recovery. Also, after 2 months post operation, the patches were resorbed completely, no trace of them being observable on the operated liver (Figure 9b).

## 4. Conclusions

The exploration of various PLA weight percentage concentrations (6%, 8% and 10%) in chloroform solutions revealed distinct outcomes, crucial for non-woven fibrous structure production. SEM analysis decisively identified the 10% PLA concentration solution as optimal, showcasing superior surface quality, a well-defined fiber structure with a mean diameter of 7.42 μm and a pervasive microporosity aspect throughout the substrate. The introduction of sulfur dioxide gas as a polymerization inhibitor within the sterile casing yielded remarkable results. This innovation assured a long enough shelf life for the patches to be used in subsequent surgical procedures with success. This aspect was supported by FT-IR analysis, which showed the bands at 3125 cm^−1^, 1615 cm^−1^, 986 cm^−1^ and 907 cm^−1^, markers indicative to the monomeric state of cyanoacrylate tissue adhesive CA, are maintained throughout the entire test period. Beyond its impact on the wound dressing’s performance, the introduction of sulfur dioxide gas also led to an improvement in the design of sterile bags, minimizing the oxygen infiltrations and leading to an enhancement in overall device functionality. As a result, the produced and tested wound dressings had excellent behavior during in vivo testing, specifically for liver resections. The peeling test on a rat model proved the excellent adhesion of the patches to the liver surface after 5 minutes in atmospheric conditions, unaffected by the forces applied to the liver sample. The force value of 1.5 N was found to be a limit for the strength of the liver tissue, as the patches remained glued to the liver.

The devices exhibited not only excellent adhesion to the parenchymal tissue, which was supported by peeling tests, but also facilitated a prompt hemostatic action.

## Data Availability

Data are available from the corresponding authors on request.
